# Recommendations for post-implementation adaptations to optimize family navigation in pediatric primary care: a qualitative study with parents and navigators

**DOI:** 10.1186/s12875-023-02072-y

**Published:** 2023-06-16

**Authors:** Julia Levinson, Emily Hickey, Plyce Fuchu, Andrea Chu, Miya Barnett, Nicole A. Stadnick, Emily Feinberg, Sarabeth Broder-Fingert

**Affiliations:** 1 Boston Medical Center, Boston, USA; 2 University of Wisconsin-Madison, Madison, USA; 3 University of Massachusetts Chan Medical School, 55 North Lake Avenue, Worcester, MA 01605 USA; 4 University of California, Santa Barbara, Santa Barbara, USA; 5 University of California, San Diego, La Jolla, USA; 6 Boston University School of Medicine, Boston, USA

**Keywords:** Health disparities, Autism, Family navigation, Adaptation, Implementation, Qualitative

## Abstract

**Background:**

Family Navigation (FN) is an evidence-based care management intervention designed to reduce disparities in access to care by providing families with individually tailored support and care coordination. Early data suggest FN is effective, but effectiveness is significantly influenced by both contextual (e.g. setting) and individual (e.g., ethnicity) variables. To better understand how FN could be tailored to address this variability in effectiveness, we set forth to explore proposed adaptations to FN by both navigators and families who received FN.

**Methods:**

This study was a nested qualitative study set within a larger randomized clinical trial of FN to improve access to autism diagnostic services in urban pediatric primary care practices in Massachusetts, Pennsylvania, and Connecticut serving low-income, racial and ethnic minority families. Following FN implementation, key informant interviews were conducted based on the Framework for Reporting Adaptations and Modifications-Expanded (FRAME) with a purposeful sample of parents of children who received FN (*n* = 21) and navigators (*n* = 7). Interviews were transcribed verbatim and were coded using framework-guided rapid analysis to categorize proposed adaptations to FN.

**Results:**

Parents and navigators proposed 38 adaptations in four domains: 1) content of the intervention (*n* = 18), 2) context of the intervention (*n* = 10), 3) training and evaluation (*n* = 6), and 4) implementation and scale-up (*n* = 4). The most frequently endorsed adaptation recommendations focused on content (e.g., lengthening FN, providing parents with additional education on autism and parenting children with autism) and implementation (e.g., increasing access to navigation). Although probes targeted critical feedback, parents and navigators were overwhelmingly positive about FN.

**Conclusions:**

This study builds upon prior FN effectiveness and implementation research by providing concrete areas for adaptation and refinement of the intervention. Recommendations by parents and navigators have the potential to inform improvement of existing navigation programs and development of new programs in similarly underserved populations. These findings are critical as adaptation (cultural and otherwise) is an important principle in the field of health equity. Ultimately, adaptations will need to be tested to determine clinical and implementation effectiveness.

**Trial registration:**

ClinicalTrials.gov, registration number NCT02359084, February 9, 2015.

## Contributions to the literature

- This is the first paper to document opinions on FN from patients and navigator perspectives
- Supports improvement and/or optimization of FN across populations and settings
- Demonstrates value of using FRAME to support ongoing, iterative knowledge exchange with patients and other stakeholders to refine, adapt, and optimize interventions

## Background

Significant racial, ethnic, and socioeconomic disparities exist in access to diagnostic evaluations and behavioral interventions for children with autism spectrum disorder (ASD). The most recent Centers for Disease Control and Prevention (CDC) data report found that in the United States Black children with ASD were less likely than White children to have a diagnostic evaluation by age 36 months, suggesting that Black children experience delays in ASD diagnosis and therefore delayed access to ASD-specific services [1]. The prevalence of ASD was also found to be lower in Hispanic children, compared to non-Hispanic children [1], suggesting that Hispanic children are identified less frequently than White or Black non-Hispanic children, and thus may be unable to access or benefit from evidence-based ASD services. Hispanic children with ASD, in particular, have been found to have difficulty accessing services because of language barriers, higher odds of not having a personal doctor, and increased difficulty receiving referrals compared with non-Hispanic, White children [2]. Additional research has shown a lower prevalence of ASD in children with lower socio-economic status (SES) compared to children with higher SES [3]. Although screening for developmental delays has increased in recent years [4] and overall ASD prevalence in the United States has increased to 1 in 44 children [1], it is clear that differential identification is affecting equitable access to services.

Targeted interventions are needed to reduce health disparities and improve long-term outcomes in obtaining access to diagnostic and treatment services for low-income, and racially and ethnically underserved children. Family Navigation (FN) is one such intervention. Emerging from principles of patient navigation for patients with chronic diseases [5–7], FN is designed to reduce health disparities by providing families with individually tailored support and care coordination [8]. FN for ASD is an evidence-based intervention [8–12] with 11 core components (e.g., training, fidelity monitoring, linguistic and cultural brokering, emotional support, care coordination, etc.) [8].

The current study was part of the implementation evaluation of a randomized clinical trial (RCT) of FN for improving access to diagnostic services and treatment for young children referred for an ASD evaluation to their health system’s Developmental and Behavioral Pediatrics Clinic [12]. FN is an intervention grounded in the chronic care model in which a navigator works closely with the family unit to support access to a specific care service over a defined period of time. FN falls under the umbrella of community or lay health worker interventions [13]. In this larger study, families in the intervention group (i.e., received the FN intervention) worked with a navigator from the time of identified concern and referral for ASD assessment to 100 days after diagnostic ascertainment. While results indicated significant reductions in time to diagnostic evaluation for all families who were randomized to FN, Hispanic families receiving FN particularly benefitted compared to Hispanic families (hazard ratio 2.81 for Hispanic children reaching diagnostic resolution with FN compared to non-Hispanic children who received FN) who did not receive the intervention (citation removed for masked review). At the same time, families who received FN in Massachusetts and Connecticut had a significant reduction in time to diagnosis; families who received FN in Pennsylvania did not. These findings indicate that FN is effective, but only in certain settings, and differentially based on the population served.

As evidence-based practices (EBPs) such as FN are disseminated and scaled-up, modifications to increase fit to specific populations or contexts are frequently made – both as adaptations, which are purposeful and planned, and as unplanned changes that occur naturally in response to challenges during implementation [14, 15]. Research has shown that adaptations to increase fit to local contexts and cultures, especially for racially and ethnically diverse populations, can improve engagement, acceptability, and clinical outcomes [15–18]. For example, a recent study comparing two pilot trials of FN found improvements in study recruitment, patient satisfaction with care, and completion of diagnostic assessment in the second pilot after implementation of a number of adaptations, including lengthening the follow-up period and changing the referral site [10]. However, to our knowledge no other studies have examined adaptations in larger scale randomized trials of FN. As FN interventions for children with ASD become more widespread, an adaptive approach to program design and implementation is crucial to ensure best practices and equitable benefits for families [19].

To best adapt FN to maximize effectiveness for low-income, racially and ethnically underserved families with children with ASD, stakeholder perspectives must continue to be taken into consideration [20]. Therefore, the aim of this study was to document patient and FN opinions about FN, and understand adaptations that may be needed to improve future implementation. To our knowledge, this is the first paper to analyze patient and navigator opinions about FN, along with considerations for improvement. Although FN is designed to improve access to care for the family unit, it was not initially designed with rigorous stakeholder input. Thus, these findings hold value to the field to refine the intervention with a more user-centered approach.

## Methods

### Study rational and framework

This study was approved by the Boston University Medical Campus and Boston Medical Center Institutional Review Board. Using an iterative qualitative design, the current study aimed to capture the voices and experiences of parents of children with ASD who received FN during their diagnostic journey, as well as the perspectives of navigators who worked with families. Interview guides and analysis of the qualitative data (key informant interviews) were developed from the Framework for Reporting Adaptations and Modifications-Expanded (FRAME) [15]. The work presented represents an advancement of this implementation science framework by extending beyond the provider perspective to also include the consumer voice [21]. In addition, we draw upon recommendations for designing implementation studies through a health equity lens to understand different patient characteristics and structural factors that impact equitable implementation [19]. Drawing from the experiences of parents of children with ASD and family navigators, the current study aimed to answer the following question: How should FN be adapted for dissemination and to ensure equity in both access to FN and ASD diagnosis and treatment across underserved populations?

### Context

The current study was part of an implementation evaluation of a RCT that took place in 11 urban primary care sites in 3 cities. Sites are described in detail in our previous publication [citation removed for masked review]. In short, primary care and developmental pediatrics clinics were all affiliated with academic medical centers in their geographic region. Although demographics varied by city, all sites served culturally and linguistically diverse, predominantly low-income populations. In the larger study from which participants from the current study were drawn, 339 parents or legal guardians with children between the ages of 15 and 27 months who were determined to be “at-risk” for ASD through screening in standard primary pediatric care were randomized to receive either a FN intervention or conventional care management control. Family navigators received training in topics such as ASD management, community resources, and motivational interviewing. Navigators met with families for a minimum of three standardized visits at critical timepoints in the diagnostic process. Beyond these visits, the intervention allowed for flexibility and tailoring to family preferences and needs. Families worked with the navigator or care manager until 100 days after receipt of a formal diagnostic evaluation. This study was approved by the hospital-based institutional review board. This paper follows the COREQ guidelines for qualitative research.

### Data collection

Parents were invited to participate via phone call from the study team. Navigators were invited to participate via email solicitation. Parents and navigators were each compensated for their time with a $50 gift card. Informed consent was obtained from all parents included in the larger trial and parents were given the option to opt out of the interview. Informed consent was obtained from navigators upon recruitment to the current study. All procedures were in accordance with ethical standards of the institution [masked for review].

All parent interviews and 6 of the 7 navigator interviews were completed by phone and audio recorded by one of three of the authors who were trained in qualitative interviewing (JL, PF, and SBF). The final navigator interview was completed and recorded in person. Interviews were completed after families reached diagnostic ascertainment. Time between termination of FN and parent interview ranged from 4 months to 3 years, with the majority of interviews conducted within the first six months after intervention completion (20 of 21 were conducted within 6-months). One interview was delayed due to initial difficulty reaching the family. All recordings were professionally transcribed, and the four interviews conducted in Spanish were also professionally translated. Transcripts were checked for accuracy by bilingual study staff (JL, PF, and AC). Recordings ranged in duration from 28 to 51 min. Bilingual members of the study team reviewed all transcriptions and translations for errors. Transcripts were analyzed in English.

### Qualitative approach and analysis

Grounded in methods common in implementation science, the qualitative approach utilized in this study was targeted and practical, and designed for rapid dissemination of findings [22]. Semi-structured qualitative interview guides (Appendix 2) for parents and navigators were developed using components from FRAME, an implementation framework used to characterize modifications to interventions [15]. Interview guides were designed to assess changes or adaptations to the intervention that parents or navigators implemented, witnessed, or reported a desire to see. For example, the following question was included in both the parent and navigator interview guide: “If you could change anything about family navigation, what would you change?” Following the lead of the parent or navigator, interviewers were guided to ask probes to specify who the modification should be for, what exactly should be changed, and the reason for the change. Interviews were conducted until data saturation was reached.

FRAME was also used to create transcript summary templates that would enable a rapid analytic approach focused on specific actionable findings [22–26]. We used an iterative design, which means we were continually updating our interview questions as we collected data in order to refine our data collection tools and select our framework. Based on this method, we selected FRAME [15] and thus recommendations were organized and assessed using FRAME. Rapid qualitative analysis is a streamlined approach to qualitative analysis designed to be less resource intensive than traditional qualitative analysis, thus requiring a shorter timeframe. In this study, two of the authors (JL and PF) independently coded all transcripts using the transcript summary templates. Any conflicts were discussed with a senior member of the team (SBF) until consensus was reached. In line with rapid analysis methods, matrix analysis was used to organize and assess recommendations in Microsoft Excel [22, 27]. This strategy allowed for a logical representation of the data that was consistent with our structured approach to analysis anchored to the FRAME [15]. All recommendations made by parents or navigators were included in analysis, including both recommendations for adaptation to the intervention (henceforth referred to as “true adaptations”), and recommendations that reflected existing components of the intervention. Results are below, and expanded in Appendix 1.

## Results

### Participants

Parents or legal guardians were eligible for this qualitative study if: (1) they were randomized to and received FN; (2) they spoke English or Spanish; and (3) their child received a diagnosis of ASD after their formal diagnostic evaluation. Parents were purposively sampled from the larger sample of parents participating in the RCT. All family navigators who worked with at least one family as part of the larger RCT were eligible for the navigator interviews. Parents were selected based on navigator nomination of parents who might be most interested in a post-trial follow-up interview. We asked navigators to recommend families who they felt had a variety of experiences with FN (both positive and negative; (participant IDs follow quotes). Although we used this strategy in an attempt to garner a variety of opinions, we recognize this may be a source of bias in our population, as FNs may have preferentially recommended individuals who they thought would report a positive experience.

As shown in Table 1, 21 parents and 7 family navigators were interviewed. This included 8% of parent participants, and 100% of navigators included in the larger study. All parents and navigators provided verbal consent to participate in the interviews. Demographics of parents and navigators were similar to those of the larger study. Thirty-eight percent (*n* = 8) of parents were Hispanic, 48% (*n* = 10) were non-Hispanic, Black, and 14% (*n* = 3) were non-Hispanic, White. Forty-three percent (*n* = 9) were born outside of the United States. Eighty-one percent (*n* = 17) opted to be interviewed in English and 19% (*n* = 4) chose to be interviewed in Spanish. Eighty-six percent (*n* = 18) of parents were high school graduates and 90% (*n* = 19) were enrolled in public insurance. Mean parent age at the time of the interview was 35.8 years (SD = 6.8) and mean child age at interview was 4.2 years (SD = 1.5). Sixty-two percent (*n* = 13) of children were male. Matching the larger study population in which 90% of participating parents were mothers, 20 (95%) of the parents interviewed were biological mothers; 1 parent was a biological father. All 7 navigators interviewed were female, as all navigators in the larger study were female. Twenty-nine percent (*n* = 2) of navigators were bilingual (1 English/Spanish, 1 English/Haitian Creole). Twenty percent (*n* = 2) were Hispanic, 29% (*n* = 2) were non-Hispanic Black, and 43% (*n* = 3) were non-Hispanic White. Seventy-one percent (*n* = 5) of navigators had bachelor’s degrees and 29% (*n* = 2) had master’s degrees.

**Table 1:** Participant characteristics

	**n (%)**	
	**Parents (** ***n*** ** = 21)**	**Navigators (** ***n*** ** = 7)**
Site		
Boston	8 (38)	3 (43)
New Haven	4 (19)	1 (14)
Philadelphia	9 (43)	3 (43)
Sex		
Male	1 (5)	0 (0)
Female	20 (95)	7 (100)
Race/ethnicity		
Hispanic	8 (38)	2 (29)
Black, non-Hispanic	10 (48)	2 (29)
White, non-Hispanic	3 (14)	3 (43)
Other, non-Hispanic	0 (0)	0 (0)
Born outside the United States	9 (43)	-
Preferred language		
English	17 (81)	-
Spanish	4 (19)	-
Highest level of education completed		
Less than a high school degree	3 (14)	0 (0)
High school degree or GED	10 (48)	0 (0)
Some college	4 (19)	0 (0)
Associate’s degree	3 (14)	0 (0)
Bachelor’s degree	1 (5)	5 (71)
Master’s degree	0 (0)	2 (29)
Insurance		
Public insurance (Medicaid)	19 (90)	-
Other	2 (10)	-
Parent age at interview, mean (SD), years	35.8 (6.8)	-
Child age at interview, mean (SD), years	4.2 (1.5)	-
Child gender		
Male	13 (62)	-
Female	8 (38)	-

### Themes

The majority of parents and navigators mentioned benefits of FN. Multiple parents cited feeling more confident and emotionally supported as a result of working with their navigator, and that they felt the navigator made the process of moving from concern for ASD to engagement in services easier than it might have been otherwise. Nonetheless, 38 recommendations for adaptations to FN emerged. Of these recommendations, 29 represented adaptations to the intervention itself and the remaining nine recommendations reflected challenges in (1) training/supervision of navigators, (2) hiring navigators, and (3) integration of the intervention into the healthcare system. Twelve recommendations were unique to parents, 15 were unique to navigators, and 11 overlapped between the groups.

Using FRAME, recommendations were assessed for what should be modified, the level of delivery at which modifications should be made, consistency or inconsistency with fidelity to the intervention, and the goal for modification (Table 2) [15]. Recommendations for adaptations to content were most commonly suggested (47%, *n* = 18), followed by those to context (26%, *n* = 10), implementation and scale-up (16%, *n* = 6), and training and evaluation (11%, *n* = 4).

**Table 2:** Suggested adaptations organized by FRAME domains [15]

	**No. %**
Adaptation or recommendation for fidelity optimization?	
Adaptation	29 (76)
Recommendation for fidelity optimization	9 (24)
*WHAT* should be modified?	
Content	18 (47)
Context	10 (26)
Implementation and scale-up	6 (16)
Training and evaluation	4 (11)
At what *LEVEL OF DELIVERY*?^a^	
Family navigator	31 (82)
Individual family	19 (50)
Supervisor/program team	15 (39)
Clinic	9 (24)
Network system/community	7 (18)
State	5 (13)
Relationship to fidelity (if recommendation were implemented)?	
Fidelity consistent/core elements preserved	23 (61)
Unknown	11 (29)
Fidelity inconsistent/core elements changed	4 (11)
What would be the *GOAL*?^a^	
Improve effectiveness/outcomes	13 (34)
Improve fit with recipients	12 (32)
Increase reach or engagement	11 (29)
Address cultural factors	9 (24)
Increase satisfaction	7 (18)
Increase retention	4 (11)
Improve feasibility	1 (3)
Reduce cost	0 (0)
**Content recommendations only (** ***n*** ** = 18)**	
What is the *NATURE* of the content modification?	
Adding elements	8 (44)
Tailoring/tweaking/refining	3 (17)
Optimizing fidelity^b^	2 (11)
Spreading	2 (11)
Changes in packaging or materials	2 (11)
Lengthening/extending	1 (6)
**Context recommendations only (** ***n*** ** = 10)**	
Contextual modifications made to which of the following?	
Personnel	7 (70)
Setting	3 (30)
Format	0 (0)
Population	0 (0)

^a^Recommendations address multiple categories, thus proportions add up to more than 100%

^b^These two content recommendations represent recommendations for fidelity optimization

### Content adaptations

As guided by domains of FRAME [15], *content* recommendations addressed changes or additions to key components of the intervention. The nature of these recommendations fell into the FRAME categories of *adding elements*, *tailoring/tweaking/refining*, *spreading (breaking up session content over multiple sessions)*, *changes in packaging or materials*, and *lengthening/extending (pacing/timing)*.

Parents and navigators suggested *adding* new navigator responsibilities. For example, six parents suggested that navigators could either connect parents to more in-depth educational resources about ASD and parenting children with ASD or offer this education themselves. One parent said, “Just like when you have a newborn, I think [navigators] should have some type of classes for how to deal with kids that have autism” (4). Parents and navigators also suggested *adding* more active navigator follow-up with parents. For example, they suggested that navigators educate parents on how to access services and navigate the system on their own; meet with the developmental behavioral pediatrician (DBP) prior to the diagnostic visit to enable the navigator to better prepare the family; connect parents to other parents of children with ASD; coordinate/consolidate services for families involved in multiple programs; attend all DBP visits with families; and connect parents to mental health therapists as needed. Parents suggested that navigators *tailor* the communication type and amount of communication to meet specific parent preference: “A navigator needs to know their family and if the family wants them to be just showing up randomly or calling and trying to show them new things unasked, then that’s good. But if they don’t, and they want to go day-to-day if they need something then they can call… knowing the family that they’re working with and knowing what that family prefers, how that family prefers to work with them” (3).

Parents also made recommendations to *refine* the intervention. A parent of five children suggested that navigators offer support for siblings of children with ASD: “Maybe a little bit more on how to better explain things to [my other children], ‘cause they kind of get it. I have a 6-year-old actually who just really is trying to understand” (7). Parents also suggested that navigators should work to de-stigmatize ASD: “That additional person there to help you out through the process just makes you more comfortable with the actual diagnosis and, you know, accepting that there is nothing different, I guess, with your child and the diagnosis” (20).

Parents and navigators recommended *spreading* the FN intervention across a longer period of time than specified in the larger RCT to allow for FN at challenging transition points, such as when children age out of early intervention (EI) services (usually at age 3) and must initiate services elsewhere. One parent said, “If my time was split between her getting diagnosed and going into [EI] and then doing that transition again at the end of 2-year-old to three – say a month or two before that, and then maybe two or three months after she’s [enrolled in services] officially. I would have really appreciated that because…– it was just too much” (3). This recommendation represents a departure from the timeline of the larger study, in which families only worked with navigators from the time of identified concern for ASD through 100 days after diagnostic ascertainment.

One parent suggested *changing the dosage* of the intervention from three core visits with the navigator to monthly check-ins: “Once a month just call the people that have autism – you know, they might have a lot of questions. Because usually when you try to call your doctors, it’s hard to get them on the phone (…) If you have a Family Navigator that would be calling every month you can kind of tell them certain things and they help you out” (19). The goal of this adaptation might be to improve fit with recipient needs and would also involve changes at the supervision team, navigator, and family levels of delivery.

*Changes in packaging or materials* (e.g., introduction to the navigator) were also suggested. Parents and navigators suggested that the primary care physician (PCP) make a real-time introduction to the navigator. One navigator specifically suggesting a warm handoff: “Let’s say once [families are] referred, the doctors say, ‘Okay, so I’m gonna refer you to DBP and I’m just gonna connect you to a navigator who’s gonna help you throughout the process. She’s gonna come into the room’” (11). On the other hand, one parent suggested that PCPs should not make the introduction to the navigator: “I personally don’t think I would prefer [being introduced to the navigator by my PCP] because I might be, you know, getting – I might be having a lot going on as is with the appointment” (2).

Many participants endorsed *lengthening/extending* the time navigators work with families past the 100 days following diagnostic ascertainment. A parent of a 6-year-old child said, “As the kids – as they grow up – they give you changes. So you expect new things coming up and you’re not familiar with this new behavior, so you don’t know how to handle it” (19).

The remaining content recommendations reflected tailoring existing components of the intervention and included: ensuring sufficient supervision of navigators, especially for difficult cases; and improved communication between the PCP, the DBP, and the navigator. As one navigator said: “I think incorporating the family navigator into the entire process. You know, having more communication with the primary care doctors, having the family navigator be a part of that medical team, and keeping them. They can be a link between the family and the organization itself because the family might say something to the navigator that they wouldn’t necessarily mention to their doctor. Having that constant communication, having that network of people together. I think it could really work in the best interest of the families if there’s that ongoing communication and embracing family navigation as part of another medical service that can be provided” (8). Both high quality supervision that includes case review and ongoing trainings, as well as effective communication between the PCP, the DBP, and the navigator are both core components of FN so these two adaptations are aligned with fidelity optimization.

### Context adaptations

Context adaptations addressed changes to the delivery of FN in terms of *personnel* and *setting*. Navigators suggested that future navigators have an educational background in social work or psychology and experience working in home settings. In addition, navigators recommended special attention to the transition process (e.g., done in-person) in cases when there is staff turnover. Two parents and one navigator suggested that navigators should have personal experience with ASD. One parent said, “I feel like people underestimate the value of personal experience. So, if you’re working with somebody [who] has a family member with autism or works with children with autism, that’s really invaluable in the process (…) You can just kind of tell when people don’t know what they’re talking about. So I feel like it really helps when somebody is understanding because of personal experience” (19).

Recommendations also emphasized the need for increased integration of FN into existing healthcare and service systems. For example, parents and navigators suggested that FN should be integrated and/or co-located into primary care, which was beyond the scope of the original FN intervention design. A parent said, “[Families] trust their doctor better than anybody. So when the doctor refers them to somebody, they will take it seriously – more serious than if it was anybody else” (1). Another parent suggested that FN should exist across healthcare systems and hospitals.

The remaining recommendations for changes to context reflect existing components of the intervention. Parents and navigators suggested that navigators be bilingual/bicultural and come from the same community and local culture as the families they serve. One navigator said, “I’ve had some moms say to me…point out to me my whiteness…my master’s degrees …and I totally understand and I… I think I was able to get past that, (…) but I think that ideally it would be someone more in the community” (2). Parents and navigators also suggested that navigators should quickly connect with families following referral to FN and navigator home visits should be offered.

### Training and evaluation

Recommendations designated as training and evaluation modifications focused on improving support for navigators. These recommendations were predominantly made by navigators and represented recommendations for fidelity optimization. Navigators suggested more training on behavior change strategies such as motivational interviewing (MI): “I think probably – yeah, [I could have been better prepared by] getting more MI skills. I think problem solving skills too… Because you’re working with different families, so obviously the situations that the families are going through is a bit different from one another” (4). Navigators also suggested more training on local service systems: “Since I was in [other state] coming to Massachusetts, this is something different. So, probably [supplemental security income (SSI)] will be different from how [other state] SSI is. So, probably the different public benefits and having applied for them and the eligibility criteria might be different. So, maybe in that sense I hope – I wish I would’ve gotten more training in that sense – on the social services” (2).

### Implementation and scale-up

Recommendations categorized as implementation and scale-up adaptations emphasized increasing access to FN by expanding eligibility criteria and improving publicity about FN availability. The recommendations all represented true adaptations to FN.

Three parents and one navigator suggested expanding eligibility to families with children with other intellectual or developmental disabilities. A parent said, “I think [the navigator] helps a lot of people (…) At any point we need their help, but not only for children with autism, but also for children who are deaf-mute and so on. I think it would be very helpful for us parents with children like that” (11). Thirteen parents and one navigator recommended increasing the reach of the intervention in general. A parent said, “I don’t care if it’s autism, cancer, whatever. I feel like any type of family that is dealing with some type of devastating news about their child would benefit from a family navigator” (19). Two parents and one navigator suggested specifically expanding FN for families who do not speak English and/or are undocumented. A Spanish-speaking parent said, “In the group my son was sent to, I met several moms who didn’t know how to go to therapy in [city]. They didn’t know how to go or what to do. And they spoke Spanish. So, people like that could receive guidance and help” (10).

Multiple parents made recommendations for disseminating FN through focused marketing or publicity on media platforms such as local television. One parent said, “I think maybe there should be flyers… or more broadcast, like maybe on TV… for somebody who don’t know or haven’t went to a clinic and know that it’s out there to have help from them” (1). Another parent suggested allowing parents to refer other parents who might benefit from navigation: "I think that improving the program would mean to check if there are more people who need it and get in touch with them. Because some of us need it but we don’t know about it. (…) If you give us permission to talk about them, I would be one of those people who could tell other people about the program" (5).

## Discussion

### Key findings

The current study investigated the recommendations of 21 parents of children with ASD and 7 navigators who participated in a RCT of FN. Through an iterative qualitative study design, recommendations for improvements to FN were elicited from parents who received FN and navigators who provided the intervention in the trial. This study builds upon prior research evidence to support FN among diverse families seeking to achieve diagnostic resolution for their child with ASD [9–12]. We provide concrete areas for the adaptation and refinement of the FN intervention based on parent and navigator experiences.

Parents and navigators identified 38 recommendations for adaptations to the content (*n* = 18), context (*n* = 10), implementation and scale-up (*n* = 6), and training and evaluation (*n* = 4) of FN. This distribution is in line with the literature: a systematic review on adaptations of evidence-based public health interventions found that content adaptations were most common, followed by context modifications and cultural adaptations [28]. Overall, recommendations highlighted a desire for expansion of FN: adding new navigator responsibilities, improving integration within the medical team, enhancing navigator training, and reaching more families for longer periods of time. This expansion would likely increase costs, a topic that did not emerge in parent or navigator interviews, but that should be of consideration in future research, and will be probed further in future data collection with family navigation researchers, organizational, and system leaders.

### True adaptations vs. recommendations reflecting existing components of FN

An unexpected finding was that nine of the 38 recommendations reflected existing components of the intervention. For example, navigators suggested adding more training on behavior change strategies, such as MI, and local service systems. This recommendation reflects the first core component of FN: “Intensive initial training to navigators on MI, navigation, problem-solving approaches, and ASD diagnostic and treatment services” [8]. Thus, the recommendation represents a need to deliver the intervention with better fidelity, rather than an opportunity to adapt FN. Although the larger RCT examined fidelity through multiple lenses, the implementation recommendations were not measured in the trial. This suggests that eliciting participant insights through periodic reflections during implementation could improve fidelity as challenges arise, rather than post-implementation [29].

While the remaining 29 recommendations represented true adaptations that participants believe may improve the intervention, future research should consider whether such changes would be consistent or inconsistent with intervention fidelity, and the potential for adaptations to add to or detract from the effectiveness of the intervention. For example, if FN were adapted to focus on care transition points, such as the initiation of EI services or the transition to school services, as suggested by parents, this would change the goal of the intervention and potentially alter what would be considered a successful outcome. If the navigator was able to get a family through the diagnostic process and enrolled in autism-specific services through EI programs, this was seen as a success in the larger RCT. However, interviews with parents a few years after study completion brought up an important point about sustained access to services. Multiple parents mentioned difficulties about transitioning from EI services to services throughout school-age. Given that EI often ends at 3 years and kindergarten usually begins at 5 years, parents of children with ASD must advocate for the child to receive services in this critical window. Thus, although these interviews identified challenges post-implementation that might have been resolved had they been identified sooner, they also uncovered additional challenges that parents faced related to the primary outcome (early identification of autism and early intervention services). Future research could thoughtfully consider multiple additional measures of success with time, or the possibility to extend autism FN to a lifespan model.

### Systems-level barriers

Given that cost can be an important barrier to navigation interventions [30], future studies might consider assessing the costs associated with these modifications and how to best expand FN while keeping a favorable cost–benefit balance. A current study, for example, is examining the most beneficial components of FN for families with young children with behavioral concerns using the Multiphase Optimization Strategy (MOST) framework [31]. It is also possible that such modifications would create additional longer-term cost saving to health care systems by balancing the added cost of FN with the cost-saving benefit of addressing developmental or behavioral concerns early, thus increasing access to supportive early intervention services, and decreasing later health-related costs. On the other hand, it is possible that making such modifications would lead to greater cost without discernable benefit, underlining the need for further refinement and testing of the intervention.

One potential modification that emerged several times was identifying characteristics of families that might benefit most from working with a navigator, often described as a “personalized” or “precision” approach [32]. For example, one parent suggested that they did not need additional support of the navigator. This parent had family members with ASD and was familiar with the symptoms, so although they had a positive view of the intervention, they felt that it was not necessary for them. Another parent suggested that members of her predominantly Spanish-speaking community might experience additional benefit from the intervention. Adapting FN implementation to engage a targeted approach – for example focusing on families with limited knowledge of child development and/or ASD, or whose native language is not English – might improve fit between the navigator and the family, helping to overcome implementation challenges, and creating a more cost-effective intervention.

### Specific recommendations for intervention adaptation

The model of FN for autism being studied in this paper aims to support parents in identifying and overcoming barriers to reaching diagnostic ascertainment, as well as in other challenges that might arise during this time period [8]. One of the most common recommendations from both parents and navigators, however, was to lengthen the intervention beyond 100 days post diagnostic ascertainment. Multiple parents mentioned challenges that outlasted diagnostic ascertainment. One parent said, “I’m very grateful to have been able to work with her. And unfortunately (…) I wish I still could because there are things right now that I want to – I’m not too certain about and I want to have more clarification but unfortunately, I don’t have her as a coordinator anymore” (17). This demonstrates the logistical challenges that families face post-diagnosis and suggests navigation might mitigate some of these challenges. Future research may consider identifying for whom an additional time period for FN would be most beneficial.

These findings are particularly important in the context of ASD considering the amount of parenting stress related to having a child with ASD, stigma, and other difficulties families of children with ASD face as they enter school and navigate the education system [33–35]. Research shows that parents of children with documented concern for ASD experience higher levels of stress than parents of children with other developmental concerns or no concerns [36]. Qualitative evidence from the current study overwhelmingly suggested that FN was able to alleviate parent stress through emotional support.

### Context

Parents in the current study also cited hardships related to other social determinants of health including housing insecurity, lack of employment, and immigration status, and how navigators were able to help in these areas. Hardships described by parents reflect the broader need for support for parents of children with disabilities. They also reflect an ongoing discussion about the importance of navigator and/or community health worker (CHW) qualifications, which have been conflicting in the literature. Tension exists regarding whether lived experience (e.g., same culture, child with ASD) or having advanced formal training is more important for intervention success.

### Strengths and limitations

A strength of this study was its use and advancement of the implementation science framework FRAME. Although many studies capture provider perspectives on adaptation of interventions, this paper highlights the consumer voice. Additionally, rapid analysis was used for faster turnaround of results, which allowed for further investigation of adaptations. This study was also strengthened by its focus on traditionally underserved families.

A limitation of this study was that most parents were interviewed a few years after their participation in FN. As a result, multiple parents had forgotten details about their experience and in some cases, their navigator. Given that navigation is novel in many settings and navigators offer help across multiple areas of need, parental confusion is not surprising. Another limitation of this study was that parents of children who were not diagnosed with ASD in the larger study but still received FN were beyond the scope of this study and thus excluded. Future studies could examine how families who participated in an intervention focused on ASD but whose children received other diagnoses benefitted or experienced potential harm (e.g., unnecessary stigma or stress) from the intervention. This could also help us better understand how FN could be used for children with other developmental and intellectual disabilities.

A final limitation of this study was that inclusion of other stakeholder groups, such as supervision teams, healthcare providers, and clinic staff, was not feasible. Interviews with members of these other groups would likely identify new recommendations as well insight into recommendations made by parents and navigators. It is also likely that these groups might identify modifications associated with cost reduction, a key area that was not raised in this study.

## Conclusion

This study demonstrates the value of embedding study of adaptation in intervention research. Despite proven effectiveness of FN, stakeholders had many recommendations for adaptation. Recommendations made by parents and navigators have the potential to inform improvement of existing navigation programs and development of new programs in similarly diverse populations. More generally, this study shows the value in continually eliciting feedback from parents and navigators in order to refine and optimize FN efforts and improvements. This is particularly important for improving equity of the intervention across populations. This is especially important given an increased national focus on expanding the mental health through different service models, such as FN, for racially and ethnically diverse families [13]. Recently, the Biden Administration released their mental health research priorities, which included a focus on understanding how to enhance the diversity of the mental health workforce through training paraprofessionals and community health worker in evidence-based practices, which could include FN. By partnering with stakeholders, including the navigators themselves, it is more likely these interventions will fit the communities they are serving and be scalable and sustainable.

## Data Availability

The datasets during and/or analyzed during the current study available from the corresponding author on reasonable request.

## Appendix 1

**Table 3:** Recommendations for modifications

**Recommendation**	**# parent endorsements**	**# navigator endorsements**	**Level of delivery**						**Content:** nature of recommendation **Context:** type	**Theme**	**Fidelity** ^**b**^	**Goal**	**Illustrative quote**
			**System**	**Clinic**	**Program team**	**FN**	**Cohort**	**Family**					
Ensure that FNs have sufficient supervision	0	2			X	X			Better fidelity to intervention	Training & Evaluation	C	Improve effectiveness/outcomes	“It’s really hard if you are on your own, like if you don’t have anyone who can help you with difficult cases. So like someone to turn to with difficult cases would be good.” – Navigator 2
Change the dosage by offering a monthly check-in option	1	0			X	X		X	Spreading (breaking up session content over multiple sessions)	Content	U	Improve fit with recipients	“Once a month just call the people that have autism – you know, they might have a lot of questions that, you know. Because usually when you try to call your doctor’s it’s hard to get them on the phone (…) if you have a [FN] that would be calling every month you can kind of tell them certain things and they help you out.” – Parent 19
Implement more active follow-up/checking in	3	1			X	X		X	Adding elements	Content	I	Increase retention; improve effectiveness and/or outcomes	“Just once we close out with the families, I feel like the families, they’re dropped and when the study ends, I feel like, even though they had the tools to continue on their own, I feel like there could be some kind of communication with the families after the study. Just to, you know, provide some sort of reassurance or to do a, a follow-up.” – Navigator 5
Lengthen the intervention	10	3			X	X		X	Lengthening/extending (pacing/ timing)	Content	I	Improve effectiveness/outcomes; increase satisfaction	“As the kids – as they grow up – they give you changes, you know. So it’s like you expect new things coming up and it’s like you’re not familiar with this new behavior, so you don’t know how to handle it, you know.” - Parent 19
Focus the intervention at transition points, such as transition into EI or school	2	2			X	X		X	Spreading (breaking up session content over multiple sessions)	Content	I	Improve effectiveness/outcomes; maybe also increase fit with recipients	“If my time was split between her going – just getting diagnosed and going into [EI] and then doing that transition again from – at the end of the 2-year-old to three. Like say a month or two before that, and then maybe two or three months after she’s [enrolled in services] officially. I would have really appreciated that because I just – it was just too much. That was just way too much.” – Parent 3
Improve communication between doctor and FN	2	2		X		X			Better fidelity to intervention	Implementation & scale-up	C	Improve feasibility; Improve effectiveness/outcomes	“We [FNs] would let [PCPs] know when appointments were scheduled and we would let them know the results of the assessment um…for the most part that’s all I initiated with them, unless I was having trouble reaching a family and they wanted to see whether they had seen them any time recently or they had new contact information or had a different way to reach out. (…) that’s something that that’s maybe is worth improving in our project… I definitely have more communication with the DBP than the PCP. – Navigator 2
Have FN meet with DBP prior to diagnostic visit so that the FN can prep the family	1	0		X		X			Adding elements	Implementation & scale-up	U	Increase satisfaction; address cultural factors	“I think it’s best if, even if [DBPs] could do like a one on one or a meeting to let the navigator know everything that is going on before, because it will help her - it will help the peer navigator to be prepared for the result. So let’s say if the kid is diagnosed with a medical condition and the parents would not know how to handle that, but at least if she already has the heads up she can do the research and find out, “Okay, the kid is diagnosed with this, I could be able to help in this way”. And then I feel that the peer navigator can be prepared. So when the parent finds the news, she’s there. She knows what to say, how to react. And she can even start preparing the parent before they find out the result. Like not tell them, but at least… easy the situation.” – Parent 5
Have the PCP make the intro to FN	3	1		X		X		X	Changes in packaging or materials	Implementation & scale-up	C	To address cultural factors; improve reach or engagement	“The introduction always made in the hospital because that’s how I get mine. I think it’s okay to let the doctor do it. It’s better because some of the people, they trust their doctor better than anybody. So when the doctor refers them to somebody, they will take it seriously – more serious than if it was anybody else.” – Parent 1
Don't have the PCP make the intro to FN	1	0				X		X	Changes in packaging or materials	Implementation & scale-up	U	Improve reach or engagement	“I personally don’t think I would prefer [being introduced to FN by my PCP] because I might be, you know, getting – I might be having a lot going on as is with the appointment.” – Parent 2
Connect parents to other parents in similar situations	1	0				X	X	X	Adding elements	Content	C	Increase satisfaction; address cultural factors	“I think [parents] would like to connect more with other parents with this issue. Because I mean that’s what I’m going through, what they’re going through with… So they kind of feel normal. Like I know like, you know, autistic kids, like that they’re normal, but when you first experience it and nobody knows anyone who’s going through it, like you really feel out of the loop and so like (…) if you can ask one of them like can you connect me or is there groups that I can go to or is there certain parks that I can go to with parents who have other children like mine who I can sit and talk with. Like the [navigators] are good for that.” – Parent 3
Emphasize holistic thinking about the families, such as interactions with siblings	2	0				X		X	Tailoring/ tweaking/ refining	Content	C	Improve fit with recipients; address cultural factors	“Um, maybe the only thing I would suggest is um, because I have, I have five kids altogether – [Child] is actually the one that we were working with and he’s the fourth. So, he has three older siblings. Maybe just a little bit more on how to, how to better like explain things to them, ‘cause they, they kind of get it. Um, I have a 6-year-old actually who just really is trying to understand. You know, but there’s only so much you can tell them.” – Parent 7
Allow option for tailoring communication type/amount based on parent preferences	3	0				X		X	Tailoring/ tweaking/ refining	Implementation & scale-up	I	To improve fit with recipients; increase retention	“A navigator just needs to know their family and know that like (…) if the family wants them to be like just showing up randomly or just calling and like trying to show them new things and like just unasked, then that’s good. But it also if they don’t, and they want to go like day to day, like if they need something then they know they can call (…) like knowing the family that they’re working with and knowing what that family prefers, like how that family prefers to work with them.” – Parent 3
Have navigator coordinate or consolidate services for families that are receiving many services	1	0	X	X		X		X	Adding elements	Content	U	Improve effectiveness/outcomes; increase satisfaction	“Okay, because like for our kids we had the [navigator], but then we also had, you know, for our other kids we had like Care Coordinators and, you know, like Therapists that were working with them, and like a whole bunch of other services. And I feel like the Navigator like could be like – because even in the name, like navigator, you think that that would be somebody who like takes care of all that stuff. Because I feel like it gets a little bit much and kind of redundant when you have so many like other people who are kind of doing the same thing (…) Maybe you could have – like the Navigators could somehow like look into all the services they’re having and find a way to like help consolidate it so that it’s not overwhelming.” – Parent 7
Add formalized education about ASD and parenting kids with ASD	6	0				X		X	Adding elements	Content	C	Improve fit with recipients; address cultural factors	“Just like when you have a newborn, I think they should have some type of classes for how to deal with kids that have autism.” – Parent 4
Add education on how to get services and navigate the system so that they can continue after navigation ends	1	1				X		X	Adding elements	Content	C	Improve effectiveness/outcomes; improve fit with recipients	“So, like if there’s a way for us to actually like, you know, sit together in front of like a laptop and actually do the search together so that they’ll know how to go about getting those services, not just me doing it. Like I can be a teacher in showing them how to like apply for certain services. Like this is the website that you go to. This is the – this is where you need to click to like, you know, actually get what you need.” – Navigator 2
Have FN attend all DBP visits with family	1	0		X		X		X	Adding elements	Implementation & scale-up	C	Improve fit with recipients; increase satisfaction; address cultural factors	“I would think that [navigator] could like go with us – like where we have appointments with [child] because we have a lot of visits in the clinic, so yeah. I wish if she could like come with us in all appointments.” – Parent 4
Have FNs offer therapy/ additional emotional support for parents or connect parents to therapy services	1	0				X		X	Adding elements	Content	U	Improve effectiveness/outcomes; increase satisfaction	“It’s a hard pill to swallow sometimes, you know. And depending on the type of parents or parent that you are, because I’m a single parent there is a lot to juggle when you have other children, and you’re not working, because you don’t trust people, your child doesn’t speak, you know it’s to a whole nother level of understanding how your life is changing that you, you know you just thought you had a kid and things want to be ‘normal’. But it’s just a flip. So, it should be good that it’s suggested for the parents to get some help as well” – Parent 19
Emphasize normalization of ASD in the intervention	4	0				X		X	Tailoring/ tweaking/ refining	Context	C	To address cultural factors	“Just that additional person there to help you out through the process just makes you more comfortable with the actual diagnosis and, you know, accepting that there is nothing different, I guess, with your child and the diagnosis.” – Parent 20
Make transition between navigators smoother, maybe through in-person meetings	0	1				X			Personnel	Implementation & scale-up	C	Increase retention; increase satisfaction	“I was able to give like an introduction, told them like who I am and like basically my role in this – in working with them. But I just feel like – I don’t know, I just feel it should be a better transition not only introduction. And just like – I don’t know. I just – having also more communication, not only through like phone, but in person so that we can like build that rapport and just like let them know that even though your prior navigator had left, like this person is here to like help you and give you that same support that the person prior had given you.” – Navigator 2
Integrate FN into primary care	4	1		X		X			Setting	Context	C	Improve reach or engagement	“There’s no way for [parents] to ask for [FN] if they don’t know about it. So maybe – maybe primary cares should be mentioning it to like, you know, people that qualify to kind of to have such a thing, you know, like yeah. So I think maybe something that should kind of mention to them if they are interested. It’s not something, you know, like if – there’s some people that might like it, you know, but they don’t know about it. So there’s no way they will be – you know – they will request for it. Yeah, so I think that’s what it should be.” – Parent 19
Broaden FN across healthcare systems and hospitals	1	0	X	X	X				Setting	Context	U	Improve reach or engagement	“[FN] should be all over the place… Meaning that it should be available for every pediatrician in the city, in the whole state, it should be available. It shouldn’t be just in Children’s Hospital. It shouldn’t just be there. It should be all over because there are families all over who have autistic children and some who are in denial that their child is even having these particular learning disabilities, like that they’re having these issues.” – Parent 17
Quickly connect with families after referral to FN	0	1				X			Personnel	Content	C	Improve reach or engagement; increase retention	“I like didn’t rush to meet with them and so I think…and this also had to do with holidays and I think it kind of got lost who I was because we didn’t pick up the first two times. I think there was something major going on in her life at the moment, like there was someone in the family in the hospital and so, there was probably several circumstances, and part of me was like I feel like I don’t need to be involved right now so, I’m going to let her deal with those things but then minus time to start getting ready for the intake and I can, kind of, make contact but um we…we had some strange communication for a couple of weeks and then we kind of rush to get everything done before the intake.” – Navigator 2
Offer home visits with the navigator	2	0				X		X	Setting	Content	C	Improve fit with recipients	“I mean it would be convenient for them to you know come out to the parent’s house, it would be cool. You know to see the parent you know having to change the routine and take your child with these issues too, you know if you don’t have a babysitter or whatever the case may be like, and I know it’s not always required for you to bring the child, but yeah, it would be nice if they could just come to you.” – Parent 19
Hire navigators with personal experience with ASD	2	1			X	X			Personnel	Training & evaluation	C	To improve fit with recipients	“I feel like people underestimate the value of maybe personal experience. And so if you’re working with somebody like has a family member with autism or works with children with autism, like that’s really invaluable in the process because then they’re not going to, you know … I don’t know. You can just kind of tell when people don’t know what they’re talking about. And when, you know, so I feel like it really helps when somebody is understanding because of personal experience.” – Parent 19
Hire bilingual/ bicultural navigators	1	3			X	X			Personnel	Training & evaluation	C	To address cultural factors; to improve fit with recipients	“Many times you can’t get someone who speaks the same language so it’s going to take longer for them, and perhaps you want to ask something, but if they don’t understand you 100%, they won’t provide the same kind of help.” – Parent 11
Hire someone from the community or the same culture	0	4			X	X			Personnel	Training & evaluation	C	To address cultural factors; to improve fit with recipients	“I’ve had some moms say to me…point out to me my whiteness…my master’s degrees um…and I totally understand and I…I don’t think it was like…I think I was able to get passed that, as like being a barrier for their…worked in this…I used to work at WIC and [Community Health Center], and I did [program]. So, that’s like what they’ve always done so, it’s not like I’m comfortable with that, but I think that ideally it would be someone more in the community.” – Navigator 2
Hire navigators that have experience going into families' homes	0	1			X	X			Personnel	Training & evaluation	C	To improve fit with recipients	“We’re going into people’s homes, um so I think just really having navigators who are comfortable with that role, comfortable going in there um and meeting the family where they’re at, and just kind of helping them move forward from there. So I think that’s important to hire the right type of people who are committed to this job.” – Navigator 4
Hire navigators with background in social work or psychology	0	1			X	X			Personnel	Training & evaluation	C	Improve effectiveness/outcomes	“It’s important to have a background in social work or psychology just to have that foundation um of understanding um people and how people interact um their needs, and culture and exploring that.” – Navigator 4
Increase access to FN for similar families	13	1	X				X		n/a	Implementation & scale-up	U	Increase reach or engagement	“I feel like any – I don’t care if it’s autism, cancer, whatever. Like I feel like any type of family that is dealing with some type of devastating news about their child would benefit from a [FN].” – Parent 19
Increase access to FN for families who do not speak English and/or are undocumented	2	1	X				X		n/a	Content	C	Increase reach or engagement	“In the group my son was sent to, I met several moms who didn’t know how to go to therapy in [city]. They didn’t know how to go or what to do. And they spoke Spanish. So, people like that could receive guidance and help.” – Parent 10
Increase access to FN for families with children with other disabilities	3	1	X				X		n/a	Content	U	Increase reach or engagement	“A person like [navigator] would help any type of families living with children with problems; not only like [ASD], but also with other issues, because not all of us have the same problems or the same type of children. So I think that in terms of the help she provides, I think she helps a lot of people… how could I put it? At any point we need their help, but not only for children with autism, but also for children who are deaf-mute and so on. I think it would be very helpful for us parents with children like that.” – Parent 11
Allow parents to refer other families	1	0	X				X	X	n/a	Implementation & scale-up	U	Increase reach or engagement	“I think that improving the program would mean to check if there are more people who need it and get in touch with them. Because some of us need it but we don’t know about it. (…) If you give us permission to talk about them, I would be one of those people who could tell other people about the program.” – Parent 5
Work with clinicians to promote FN	1	0		X				X	n/a	Implementation & scale-up	U	Increase reach or engagement	“I think that regarding the doctors, they make a good team, because that’s where you will find more people like us waiting for help.” - Parent 3
Use publicity tools such as flyers or TV commercials	3	0	X	X				X	n/a	Implementation & scale-up	U	Increase reach or engagement	“I just think that it should be – if it wasn’t for me going to the clinic I wouldn’t know anything about it. So I think like maybe there should be like flyers… or like more broadcast, like maybe on TV and stuff… for somebody who don’t know or haven’t went to a clinic and know that it’s out there to have help from them.” - Parent 1
Make sure navigators understand local services systems	0	2			X	X			n/a	Context	C	Improve effectiveness/outcomes; increase fit with recipients	“Since I was in [other state] coming to Massachusetts, like this is something different. So, like certain, you know, probably [supplemental security income (SSI)] will be different from how [other state] SSI is. So, like probably like the different public benefits and having applied for them and the eligibility criteria might be different. So, maybe in that sense I hope – I wish I would’ve gotten more training in that sense. Like on the social services.” – Navigator 2
Make sure navigators receive orientation and training that includes MI	0	3			X	X			n/a	Training & evaluation	C	Improve effectiveness/outcomes	“I think probably – yeah, [I could have been better prepared by] getting more MI skills. Also like I think it’s also like problem solving skills too… Because like – yeah, like you’re going to – you’re working with different families, so obviously some – the situations that the families are going through is a bit different from one another.” – Navigator 4
Lengthen the training period for navigators	0	1			X	X			n/a	Training & evaluation	C	Improve effectiveness/outcomes	“I think just like a longer period of training, not just 1 week or 2 weeks (…) And like especially if the person doesn’t have any – like experience living with families, then obviously they would need that more of a training. But since I – obviously I was a different case since I worked the families but – yeah. I think it just – like it really depends on the situation.” – Navigator 4
Include training on reporting abuse	1	0			X	X			n/a	Training & evaluation	C	Improve effectiveness/outcomes	“I don’t know, I’m thinking hypothetically, this wasn’t my case, maybe if they see that someone is being abused or harassed, they should notice and maybe derive it or report it to the corresponding authority, I don’t know. Because that could happen.” – Parent 1

^b^ *C* Fidelity consistent, *I* Fidelity inconsistent, *U* Unknown effect on fidelity

## Appendix 2

### Parents- interview guide

***Intent statements:*** *Each section of this guide is preceded by an intent statement which explains the type of data that the section has been designed to gather. Intent statements are designed so that all interviewers focus their discussion on the same areas of interest and thus gather similar data. This will allow the interviewer to deviate from the script for a more naturally flowing conversation while also ensuring that relevant themes are being captured.*

***Interview questions:*** *There are two primary types of questions: open-ended, “lead” questions and probes which tend to be less open-ended. Both lead questions and probes are intended to guide the interviewer to gather all the data investigators are seeking. They are not meant to be used verbatim. Interviewers should consider the best way to gather the data and rephrase questions to address intents and practices.*

#### Introduction

Thank you for offering your time today! As you may remember, Family Navigation is a program that was created to help families better connect with the health care and community systems and the services available to children. Family navigators help parents and caregivers better understand a provider’s advice for their children’s health. They help families overcome some of the barriers or difficulties, like transportation issues, language and cultural differences they may face when trying to attend appointments or access services for their children. Navigators work with families throughout the developmental assessment process. After the developmental assessment process has been completed, they continue working with families for 100 days to help them access services their children may need. Do you have any questions about Family Navigation?

During this interview, we intend to discuss your experience of working with your Family Navigator. As we discussed before, this call will be recorded and de-identified. Only key study staff will have access to your interview.

Before we begin, I want to remind you of a few things:

1. You can take a break or ask to turn off the recorder at any time.
2. You are encouraged to tell me (the interviewer) if you feel uncomfortable for any reason.
3. You can ask to stop the interview at any time – you do not have to keep going if you choose not to.

Do you have any questions before we begin?

Now, I’d like to ask you questions about your experiences. Please be open and honest. There are no “right or wrong” answers to the questions I will be asking you.

**Table:**

**Part 1A: Intervention- First Impressions of Family Navigation**
***Intent:*** *The intent of this section is to hear about parents’ first impressions of family navigation. This may include information about their expectations or feelings regarding their navigator. Keep in mind that while some parents may not remember specifically their first interaction with their navigator, we still are interested in their general feelings and perceptions at the beginning of the process.* **“Please start by telling me a bit about your first experiences with your Family Navigator.”**
**Q1:** How did you first find out you would be working with a Navigator?
• Who initially told you that you would be working with a Navigator? • What did they say?
**Q2:** How did you feel when you were first referred to your Family Navigator?
• What was going through your mind when you were referred? • What did you expect to be helpful about having a Family Navigator? • What did you think would be challenging or difficult about having a Family Navigator?
**Q3:** Please tell me about the first time you spoke with your Navigator
• What did you discuss? • What made you feel comfortable or uncomfortable? • Was there anything that was confusing about the conversation? If yes, why?

**Table:**

**Part 1B: Intervention- Working With Your Navigator**
***Intent:*** *The intent of this section is to understand parents’ experiences working with their navigator. This may include perceptions of the role of the navigator, feelings throughout the navigation process, examples of how the navigator did or did not support the families, and general nuts and bolts of working with a navigator. By the end of this section, the interviewer should have a good understanding of what actually happened during the navigation period and how the parent felt about it.* **“Now let’s talk about what it was like to work with your Navigator.”**
**Q4:** Before you started the process, what did you think it would mean to have a Family Navigator?
• What role did you expect the navigator to play? • How did your expectation compare to what it was actually like having a Navigator? • [**If not already answered by previous question**]: Looking back, how would you describe the job of your Family Navigator? • What are some words that best describe your experience of working with a Navigator?
**Q5:** What did you usually discuss with your Navigator?
• Where did you typically meet with your Navigator? Why did you choose this location? Where do you think would be the best location for parents to meet with navigators?
**Q6:** How, if at all, did your Navigator help or support you during the process?
• Please give me some examples of things your Navigator did to help or support you in this process. ◦ [**If parent can’t think of anything**]: Sometimes we hear parents talk about getting help from Navigators with things like transportation to visits, scheduling appointments, or connecting to services. Other parents talk about getting emotional support from Navigators. In what ways, if at all, did your Navigator help or support you? • Can you tell me about specific instances where you felt you needed your Navigator to help you more? • What about times when you felt you needed less help from your Navigator? • How could your Navigator have better helped or supported you? • What else do you wish they had done? • **[If parent says that they received no help]:** ◦ What would you have liked help with? **▪ [If parents come up with something]**: Did you feel you could have asked your Family Navigator to help you with it? If not, why? ◦ How could they have better helped or supported you?
**Q7:** What was the most important thing that your Navigator did for you? Why?
• What was the least important thing that they did for you? Why?
**Q8:** Tell me about your experience discussing family navigation with family, friends, or other people.
• What did they say? **[Make sure to specify whether results relate to family, friends, or both.]** • How open were you to hearing their opinions?
**Q9:** Did your pediatrician or any other provider help you connect with or support you with your Navigator?
• How could they have better helped you connect with your Navigator? • What, if any, additional support was provided by the pediatrician? • How, if at all, should family navigation fit into the health care system or with your pediatric clinic?
**Q10:** Navigators work with families in different ways. They do home visits, calls, accompany families to appointments, and more. What type of interaction did you find most helpful when you worked with your Navigator? Why?
• Did this differ based on what topic was being addressed? How? • What type of interaction was least helpful? Why?
**Q11:** How did you usually communicate with your Family Navigator (for example, phone calls, texts, or other ways)?
• What type of communication do you think worked best? • What type of communication was least effective? • How often did you communicate with them? • How satisfied were you with how much you communicated with your Navigator? ◦ If you were to go through this process again, would you want to communicate with your Navigator more often, less often, or about the same amount? Would you want to communicate in the same way again (texting, calling, etc.)?
**Q12:** On a scale of 1–10, with 1 being almost nothing, and 10 being an expert, how well do you think your Navigator understood **autism and other developmental concerns**?
• Why did you choose this number? • In what ways did their level of understanding show when working with you?
**Q13:** **[If not already answered in Q12]:** On a scale of 1–10, with 1 being almost nothing, and 10 being an expert, how well do you think your Navigator understood **the diagnosis process and accessing services**?
• Why did you choose this number? • In what ways did their level of understanding show when working with you?

**Table:**

**Part 1C: Intervention- Characteristics of the Navigator**
***Intent:*** *The intent of this section is to understand what characteristics of the Navigator were helpful or not helpful for the parent. This could be actions that the Navigator took or characteristics of their personality.* **“Thank you for sharing your thoughts with us so far. Now we’re going to talk about the characteristics of a Navigator that make them successful or not successful.”**
**Q14:** What specific things did your Navigator do that made you want to work with them?
• How did this make you feel? • What things did they do that made you NOT what to work with them? Why? • Did you feel that you could trust your Navigator? Why or why not?
**Q15:** What made it easy to work with your Family Navigator? Why?
• What made it difficult? Why? • What were your friends’ and family’s reactions to you working with a Navigator? • How available or accessible was your Navigator?
**Q16:** Was there ever a time that you didn’t want to work with your Family Navigator?
• If yes: ◦ At what point during the process did this happen, such as at the beginning when you first met them or later on? ◦ Why didn’t you want to work with your Navigator?
**Q17:** What did you like about your Navigator? What did you not like?
• What did you like about their personality? What did you not like? • Was there anything your Navigator did that bothered you?

**Table:**

**Part 1D: Intervention- Challenges Associated with Family Navigation**
***Intent:*** *The intent of this section is to hear about challenges that parents faced with the diagnostic process/ accessing resources and also challenges that they faced in working with the family navigator. Allow parents to think of challenges they faced without leading them in any direction. If they mention challenges unrelated to the navigator, ask questions about how/if the navigator helped them overcome these challenges.* **“Let’s discuss some challenges that you may have experienced when working with your Family Navigator. Challenges can be anything that was hard for you.”**
**Q18:** Take a moment to think about the challenges that you experienced when working with your Family Navigator. What challenges come to your mind?
*• For each challenge mentioned by the parent, ask the following questions (only as relevant)*: ◦ How or why was this challenging for you? ◦ How, if at all, was this challenge addressed? By whom? ◦ How could this challenge have been avoided or better addressed? ◦ How long did you experience this challenge? For example, was this a short-lived challenge, or was it present for an extended period of time? ◦ How successful were you and/or your Navigator in finding a solution to the challenge? *• Keep eliciting responses until the parent can no longer think of more challenges. Try to get at least 3 challenges. If parents can’t come up with any or enough responses, try using the following questions:* ◦ Some **challenges in working with the Navigator** that we’ve heard from other parents include: lack of communication, language barriers, or an unclear understanding of the relationship with the Navigator. What kinds of challenges might you have faced with your Navigator? ◦ We also hear parents talk about **challenges in the autism diagnosis and treatment process**, such as making appointments or understanding and trusting the diagnosis of the doctor. Sometimes Navigators work with parents to address these challenges. Do these challenges make you think of anything else you may have faced?

**Table:**

**Part 2: Measuring Change over Time**
***Intent:*** *The intent of this section is to understand what parents have learned throughout the process and how their perspectives or emotions have changed over time. For example, we would be interested to know if parents are more empowered or have better coping skills at the end of the process.* **“Now that we’ve covered quite a bit about your experience with Family Navigation, I think it’s a good time to discuss changes that you may have seen over time.”**
**Q19:** How do you think your experience would have been different if you had not worked with a Navigator?
• How, if at all, has your understanding of treatments and how to obtain services changed over time?
**Q21:** On a scale from 1 to 10 where 1 is not at all and 10 is very well, how well did the Family Navigator provide you with the knowledge and skills necessary to complete this process and get connected to services?
• Why did you choose this number? • What type of assistance, if any, do you wish you could have had longer from the Family Navigator? • What else could your Family Navigator have done for you?
**Q22:** Reflecting on your experience working with a Navigator, what has been the most valuable thing you’ve learned? Why?
• Knowing what you know about going through the process, what, if anything, do you wish you had learned?
**Q23:** Doctors are trying to make it easier for children with developmental concerns to get diagnosed and treated. If you could give doctors one suggestion to make things easier for families, what would you suggest?
• How would this help? • How could this have helped you?

**Table:**

**Part 3: Autism Knowledge and Perceptions**
***Intent:*** *The intent of this section is to hear how parents understand and perceive ASD. This is not dependent on diagnosis—we want to hear about perceptions of ASD from both parents of children diagnosed with ASD and parents of children who received no diagnosis or another diagnosis.* **“Since our project is focused on children with developmental concerns, often for autism, why don’t we conclude the interview with a few questions about your thoughts on the diagnosis process and autism in general.”**
**Q24:** What, if any, diagnosis did your child receive?
• What was it like for you to receive this diagnosis? • When the doctor told you about the results of the evaluation, how did you feel about the outcome? • How did the outcome compare to how you saw your child? • How much did you agree or disagree with the diagnosis? Why?
**Q25:** On a scale of 1–10, with 1 being almost nothing, and 10 being an expert, how much did you know about autism before enrolling in this study? How much do you feel like you know now?
• What score would you say you started with, and what score would you have right now? • Why did you choose these numbers?
**Q26: [ONLY ASK PARENTS OF CHILDREN DIAGNOSED WITH ASD]** Tell me about your experience discussing autism spectrum disorder with family, friends, or other people.
• What do you say? What do they say? • What do your friends and family know about autism? • Do you think some people have the wrong ideas about autism? Why? • Do you think people treat your child differently if they know he/she has autism? Why?
**Q27:** Where do you think most people (including your family and friends) learn about autism?
• What messages do people get from the media, such as TV or the news? • What kinds of messages do people get from friends or family? • What kinds of messages do people get from health care providers, such as their pediatrician? • How, if at all, might Family Navigation change the way people think about autism?

**Table:**

**Part 4: Parent’s Recommendations Regarding the Implementation of Family Navigation**
***Intent:*** *The intent of this section is to hear parents’ perceptions about the implementation of family navigation. This includes whether parents believe a family navigator should be available to families, who would benefit more from a having a family navigator, and how family navigation should be introduced to families.* **“Now that we’ve discussed your personal experiences working with your family navigator, let’s talk about your views on having family navigation available to families.”**
**Q28:** How do you think families can benefit from working with a family navigator?
• What kind of support (or resources) do you think families would like to receive from a family navigator? What kind of support is less important for families to receive?
**Q29:** Who (or what type of families?) would benefit most from working with a family navigator?
• What type of families would need more help or support from a family navigator? What families might need less help?
**Q30:** What do you think might make it hard for families and family navigators to work together?
• Do you think there might be any harms with working with a family navigator?
**Q31:** How should families be introduced to family navigation (or a family navigator)? (e.g. in-person or via phone)
• Who should make the introduction?
**Q32:** Navigators go through training about autism, the service system, and how to help parents through the diagnostic process. In your experience with your navigator, how well trained did she seem?
• How do you think training could be improved? • Are there other things that the navigator could be trained in that you think would be helpful?
**Q33:** If you could change anything about family navigation, what would you change?
**[Following the lead of the parent, ask follow up questions to specify WHO the modification should be for, WHAT exactly should be changed, the REASON for the change, etc.]** • **[If not already mentioned]**: Other parents have mentioned suggestions in the following areas: education for parents about autism and how to deal with having a child on the spectrum, better coordination between navigators and doctors, and longer periods of navigation. What do you think of these ideas?

**Table:**

**Part 5: Concluding questions**
***Intent:*** * The intent of this section is to give the parents/caregivers the opportunity to share any additional thoughts with the interviewer. The interviewer should make sure to end on a positive note and indicate the helpfulness of data provided by the family.*
**Q33:** You have told me so much about your family’s experience. Is there anything else you would like to share about your experience with family navigation or suggestions for how to help other families in the future?

*****Conclude by thanking them for their time and willingness to share their thoughts so openly!***

### Navigator interview guide

**“Thank you for offering your time. Through this interview, we intend to discuss your perspective on family navigation and its effectiveness as an intervention. As a reminder, this call will be recorded and de-identified. Only key study staff will have access to your interview.”**

**Table:**

**Part 1: Intervention- Engagement with Families**
**“Let’s start by discussing what it’s like to engage families in navigation.”**
**Q1:** Reflect on the families you’ve worked with. In general, what do you think it means to engage with a family?
• What are some words that best describe your experiences of engagement? • Did you find that some families required more emotional support? • Any that avoided emotional support? **IF YES:** That can be challenging. How did you address that? • Did some require more guidance than others?
**Q2:** Engagement can involve different tasks for everyone. Can you think of any specific examples of things you’ve needed to do in order to engage families?
• Did this family present challenges that you haven’t had prior experience with?
**Q3:** What do you think are some of the barriers that make it more difficult to engage with a family?
• Do you think there are trends (demographically or across diagnoses) among families that present these barriers? • Which barriers do you think are most challenging to address? What has your experience been with these types of barriers?

**Table:**

**Part 2: Training and Role Description**
**“Now let’s talk about training for your role as a navigator.”**
**Q4:** Please start by telling me about your experience with training to become a navigator.
• Was the training helpful? What components of training were the least/most helpful? • Positive? Negative? Overwhelmed? • Are there specific parts of your training that proved helpful in a particular type of scenario? • Were you confident in your ability to proceed as a Family Navigator after training?
**Q5:** How could you be better supported through trainings?
• What would you change? Would you add or remove components of training? • Is there something you know now that in hindsight, you think would have helped you when first starting off? • **If YES:** Please explain this piece of advice and describe the particular event when this occurred to you; **IF NO:** skip to Part 3

**Table:**

**Part 3: Communication Across Study Personnel**
**Another critical piece to intervention success is the degree of communication across study personnel. Why don’t we discuss that.”**
**Q6:** Who would you describe as the primary person who oversees your work as a Navigator?
• How often do you communicate with that person? • By phone, email, in person? What do you generally discuss? • How often, let’s say on a weekly basis, do you communicate and/or discuss your cases? • To what extent do you feel supported by your supervisor? How could supervision be improved?
**Q7:** How would you best describe the relationship between you and the site PI? How about the sense of communication between you both?
• Do you think it’s strong? How about consistent? • **IF YES:** How so? What characteristics about this relationship stand out to you? • **IF NO:** What are some of the challenges you’ve experienced in maintaining a consistent, strong stream of communication with the PI?
**Q8:** How often do you meet with the site PI? Is this a frequent practice, or something that’s schedule as needed?
• What modes of communication do you typically use when you’re not meeting?
**Q9:** How often do you communicate with the DBP and/or necessary providers?
• How do you think communication with providers influences the effectiveness of your role as a Navigator? What, if any, are some of the challenges you’ve faced when communicating and trying to communicate with providers?
**Q10:** Who do you think you talk to most out of all study staff that you communicate with?
• Which interaction do you find to be most valued in terms of assistance and/or guidance? • Is there someone who is challenging to get in touch with and/or simply challenging to communicate with in general?
**Q11:** How would you describe the sense of communication between staff across sites?
• What do you typically discuss? • Would you say you communicate frequently? Perhaps not enough? • How do you think frequent communication across site coordinators may influence the intervention?

**Table:**

**Part 4B: Intervention- Measurement of Implementation and Sustainability**
**“Let’s move on to discussing the inner setting of the intervention.”**
**Q17:** The inner setting of an intervention is the actual space in which an intervention is carried out. What do you perceive as being the inner setting for this particular intervention? Please explain.
• Where do you feel navigation is based out of? • What part(s) or characteristic(s) of the inner setting do you think have a positive influence on day to day implementation? • What part(s) or characteristic(s) of the inner setting do you think have a negative influence on day to day implementation?
**Q18:** Where do you think your role fits within the inner setting?
• What parts of the inner setting help to support your role? • What parts of the inner setting make your role more challenging?
**Q19:** To what extent does your professional environment support programs like Family Navigation?
• Was there anything in particular that helped you to better support the implementation of family navigation? • In what ways could your professional environment better support family navigation?

**Table:**

**Part 4C: Intervention- Family Demographics and Challenges Associated with Family Navigation**
**“Let’s move on to discussing characteristics you’ve noticed across families you’ve worked with and challenges that you may have experienced as a Family Navigator. Challenges can be anything that was hard for you.”**
**Q20:** Have you noticed any trends in demographics across families that are receiving care at the clinic?
• If so, what are they? • Do you think culture plays a role in a families’ willingness to interact or engage in navigation? ◦ If yes, how, if at all, can family navigation better take culture into account? • How do you think they influence families’ engagement and/or involvement in Family Navigation?
**Q21:** Have you noticed any trends across families in receptivity to DBP referrals or navigation?
• Are there certain characteristics that you think may be indicators of receptivity, or lack of it?
**Q22:** The following are a list of things we feel may have been challenging for some families. Please let me know if and how often you’ve witnessed difficulty with any of these:
• Making or scheduling appointments • Transportation to and from appointments (or long travel distances) • Seeing a specialist • Unclear understanding of steps you needed to take • Lack of communication between provider or Family Navigator • Language barriers • Trusting the doctor’s diagnosis • Extended wait to obtain an appointment • Unclear understanding of where to go for services • Familial issues • Documentation challenges • Religious challenges - How did you help to address those challenges? - What guidance did you need to seek, if any, when faced with these challenges?

**Table:**

**Part 4: Navigator’s Recommendations Regarding the Implementation of Family Navigation**
***Intent:*** *The intent of this section is to hear navigators’ perceptions about the implementation of family navigation. This includes whether parents believe a family navigator should be available to families, who would benefit more from a having a family navigator, and how family navigation should be introduced to families.* **“Now that we’ve discussed your experiences working with families, let’s talk about your views on having family navigation available to families.”**
**Q28:** How do you think families can benefit from working with a family navigator?
• What kind of support (or resources) do you think families would like to receive from a family navigator?
**Q29:** Who (or what type of families?) would benefit most from working with a family navigator?
• What type of families would need more help or support from a family navigator? What families might need less help? • How, if at all, should we tailor the design of family navigation to meet the needs of different types of families?
**Q30:** What do you think might make it hard for families and family navigators to work together?
• Do you think there might be any harms with working with a family navigator? • How can we best avoid harms?
**Q31:** How should families be introduced to family navigation (or a family navigator)? (e.g. in-person or via phone)
• Who should make the introduction?
**Q32:** If you could improve anything about working with families, what would it be?
• What advice would you give a new family navigator about how to best work with families?

**Table:**

**Part 5: Measuring Change over time**
**“Now that we’ve covered quite a bit about your role within the intervention, I think it’s a good time to discuss changes that you’ve seen over time.”**
**Q23:** What do you think has changed within the intervention from the beginning of the study?
• What are some positive changes? • What are some negative changes? • What do these changes look like in the clinic? • Are these changes within the structure of the intervention?
**Q24:** How do you think these changes influenced the success of the intervention in particular?
• Are there individuals, both outside of and within the clinic, that you feel have become more open to the intervention over time? • How have these changes influenced your role as a Coordinator?
**Q25:** Based on the changes you’ve witnessed so far, do you feel this intervention could be sustained across settings?
• What do you feel would need to happen, both within the immediate clinical setting and systematically, for this intervention to be sustained outside of a research study?

## Abbreviations

ASD: Autism Spectrum Disorder
CDC: Centers for Disease Control and Prevention
CHW: Community Health Worker
DBP: Developmental Behavioral Pediatrician
EBP: Evidence-Based Practices
EI: Early Intervention
FN: Family Navigation
FRAME: Framework for Reporting Adaptations and Modifications-Expanded
MI: Motivational Interviewing
MOST: Multiphase Optimization Strategy
PCP: Primary Care Physician
RCT: Randomized Clinical Trial
SES: Socio-Economic Status
SSI: Supplemental Security Income
